# Emergency surgical airway experience from an Australian major trauma centre emergency department

**DOI:** 10.1186/s13049-025-01413-3

**Published:** 2025-06-12

**Authors:** Christopher Groombridge, Amit Maini, Dries Helsloot, Carl Luckhoff, Mark Fitzgerald

**Affiliations:** 1 School of Translational Medicine, Monash University, Melbourne, Australia; 2 Emergency & Trauma Centre, The Alfred Hospital, Melbourne, Australia; 3 National Trauma Research Institute, 85–89 Commercial Road, Melbourne, VIC 3004 Australia; 4 Department of Cardiovascular Sciences, Kulak University, Kortrijk, Belgium

**Keywords:** Cricothyroidotomy, Intubation, Surgical airway, Emergency front of neck access

## Abstract

**Background:**

Emergency front of neck access (eFONA) may be life-saving in the can’t intubate can’t oxygenate scenario but the frequency with which an individual emergency department (ED) or emergency physician (EP) will be required to perform this intervention is very low.

**Objective:**

Identify and describe all eFONA cases from the Alfred Airway Registry and to estimate the per clinician incidence of the procedure.

**Methods:**

Retrospective case series of all eFONA cases from the Alfred Airway Registry. Data on all intubations undertaken in the ED were collected prospectively from February 2017 to January 2025. Data on individual clinician experience of eFONA was captured by an electronic survey.

**Results:**

Of the 1805 patients intubated during the 8 years study period, 4 cricothyroidotomies were performed (0.22%) with a scalpel-finger-bougie-tube technique. All were performed outside daytime hours (08:00–18:00) and all were successfully completed by clinicians who had previously practiced the procedure on a cadaver. 75% were in trauma patients, 75% were male and 75% were performed by emergency medicine doctors. From the survey data EPs performed 24 surgical airways in 768 years of consultant-level experience.

**Conclusions:**

eFONA is a rare intervention occurring approximately once every 2 years in this trauma centre ED, and once every 32 years of consultant-level experience for the centre’s EPs. The scalpel-finger-bougie-tube technique reliable achieved a secure airway in these patients.

## Background

Emergency front of neck access is an intervention that may be life-saving, though rarely required. In the operating theatre the reported incidence is 0.006–0.16% [1]. The incidence outside the operating theatre environment is much higher on a per patient basis, estimated at 0.3–1.1% for patients intubated in the emergency department (ED) [2–4] and up to 1.6% for those intubated in the intensive care unit (ICU) [5]. However, the per clinician exposure remains rare. Moreover, improvements in airway management in the last decade, including quality improvement initiatives [6, 7], increased bougie [8] and videolaryngoscopy usage [9], make this an increasingly uncommon procedure [10].

Debate continues on the most appropriate technique to achieve emergency front of neck access, with advocates for ‘needle’ or ‘puncture’ techniques [11, 12] and ‘open’ or ‘surgical’ techniques [13]. Even the related nomenclature is a matter of continued discussion with some clinicians favouring ‘Can’t intubate can’t oxygenate (CICO) rescue’ or a variation of ‘emergency front of neck access’ (eFONA) as the main contenders [14].

Heard and colleagues report a high success rate using an algorithm centred around needle techniques, however, they acknowledge that this success is likely to rely on an extensive training program, using anaesthetised pigs, that underpins this intervention [11].

Crewdson and Lockey’s commentary [15] on a systematic review of emergency cricothyrotomy [16] provides a sober analysis of the landscape, and in our institution, we align with their view that a simple open technique using readily available equipment (scalpel and bougie) is the optimal technique for a heterogeneous cohort of critical care clinicians with varying exposure to training.

Irrespective of the procedural technique chosen, the human factors associated with the decision to perform eFONA is an important consideration and forms a significant component of the training around this procedure at our institution. Clinicians may experience task fixation, communication breakdown and cognitive overload as a consequence of the stress which is inherent to these scenarios [17–19]. Airway algorithms exist to assist clinicians with decision-making in a difficult airway scenario, examples include the Difficult Airway Society (DAS) guidelines [20] and Vortex approaches [21]. In our education program we discuss a simplified heuristic: Outside the operating theatre environment there are only three standard methods to ventilate the patient (bag-valve-mask (BVM), supraglottic airway (SGA), or orotracheal intubation). If these methods have been unsuccessful by the most experienced member of the available critical care team, and the patient’s oxygen saturations cannot be maintained above 70% then a surgical airway should be performed. It is the authors opinion that any clinician who may be in the position to supervise intubation should be able to perform an emergency surgical airway.

The aim of this study is to report the cases of surgical airways performed in an Australian major trauma centre emergency department and describe the underpinning quality improvement and education program that supports this practice.

## Methods

This case series is part of an ongoing quality improvement and education program at the Alfred hospital [6, 22]. The Alfred is one of the two level-1 adult trauma centre emergency departments (ED) in Melbourne, Australia, which sees over 70,000 patients per year, of which over 1,600 are classified as major trauma patients (Injury Severity Score > 12).

The quality improvement program was started in 2017 and includes three elements:

1. A secure online data collection tool, The Alfred Airway Registry, which emergency trainees complete for every intubation occurring in the ED.
2. An intubation checklist to ensure consistent preparation for airway management which is read aloud between the airway nurse and airway doctor.
3. An education program, which is based on the airway registry data, and includes a review of cases which were either not successful on the first attempt or cases with adverse events, particularly hypoxia or hypotension. In addition, emergency physicians have access to two procedure focused cadaver courses which teach a ‘scalpel-finger-bougie-tube’ technique (simplified description in Box 1).

**Box 1:** Summary of procedural steps for cricothyroidotomy

Procedure steps:
1. Extend the neck. Remove any cervical collar that may be present; airway patency is the primary life-saving Intervention.
2. Immobilise the larynx with your non-dominant hand, palpating for the cricoid with your non-dominant index finger. We recommend a right-hand-dominant clinician stand to the patient’s right, allowing them to hold the thyroid cartilage with their left hand.
3. Make a longitudinal incision centered on where you think the cricothyroid membrane is located. When unsure make a longer incision. An 8 cm incision commencing 3 cm above the supra-sternal notch reliably includes the cricothyroid membrane in adults [23].
4. Re-palpate the anatomy within the incision. The cricoid membrane should be easier to feel (don’t expect to be able to visually identify structures as bleeding is expected - your palpating non-dominant index finger is your ‘eye’).
5. Make a horizontal incision through the cricoid membrane extending to each side of the cartilaginous ‘mouth’ formed by the junction of the cricoid and thyroid cartilages laterally.
6. Remove the scalpel and insert your index finger (the thyroid and cricoid cartilages articulate with each other, and this space will increase in size to accept your finger).
7. Slide the bougie along the pad of your index finger into the trachea (by having your finger and bougie in the same hole at the same time you greatly reduce the risk of a false passage).
8. Railroad the endotracheal tube (ETT) into the trachea, just until the balloon is through the incision.
9. Inflate the cuff, remove the bougie and ventilate the patient ensuring an end-tidal CO_2_ trace and improved oxygen saturation.
10. Secure the tube with a braided non-absorbable suture ‘drain stitch’ (e.g. 1 Silk).

### Data collection and analysis

Every intubation undertaken within the ED is captured using a secure audit tool (Jotform), which the emergency trainees caring for the patient completes immediately after the intubation. Data collected has previously been described [6] and includes patient demographics, details of the preparation for airway management, including airway assessment, preoxygenation and induction medication, and features of the attempts at intubation, including staff involved, devices used and adverse events related to airway management.

Emergency physicians were surveyed on their own experience of eFONA with a short questionnaire: (1) How many eFONA have you personally performed in Australia? (2) How many years have you been an EP? (3) Do you keep a logbook / tally of the exact number of intubations you have personally performed?

Data were summarised using descriptive statistics with continuous variables summarised as mean and standard deviation (SD) for normally distributed variables, and median and interquartile range (IQR) in cases of non-parametric distribution. Categorical variables are presented as frequencies (n) and proportions (%). Continuous variables were assessed using the Student’s t-test, whilst skewed or categorical data used the chi-square test or Fisher’s exact test (if number in a cell was < 5).

## Results

Data collection began in February 2017 and is ongoing. This data set is from the registry’s inception to January 2025 and includes 1805 patients intubated, approximately 19 per month. Of the cohort, 4 patients underwent a surgical airway (0.22%).

Table 1 compares key results for these patients with the overall cohort of patients intubated in this emergency department over the last 8 years.

Patients undergoing surgical airways had a very similar age, sex and weight distribution to the overall cohort. The majority (56.8%) of all airway management at this institution occurred outside of daytime hours (18:00 to 08:00), and all surgical airway cases occurred out of hours during the study period. This institution has 24-hour consultant anaesthesiology coverage and are included in the ‘trauma callout’ where they undertake, or supervise, the airway doctor role for these cases. A consultant anaesthesiologist was present for all the surgical airway cases in this series. 24-hour consultant emergency physician coverage was implemented in 2022 and there have been no surgical airway cases since then.

Hypoxia (saturation < 93%) at the time of decision to intubate was present in 25% of the surgical airway cases compared with 13.49% of the overall cohort (*n* = 1 vs. *n* = 243, *p* = 0.441). Desaturation during attempts at airway management was more common (75% vs. 10.49%, *p* = 0.004) as was right mainstem bronchus intubation (50% vs. 1.17%, *p* < 0.001). Rates of hypotension (0% vs. 9.88%, *p* = 0.66) and bradycardia (0% vs. 0.28%, *p* = 0.99) related to airway management was similar.

**Table 1:** Characteristics of airway management cases from the Alfred airway registry

Variable	Surgical Airway	All intubations
	*n* = 4	*n* = 1801
Age (years), median (IQR)	48.5 (38.0–52.0)	43.0 (30.0–60.0)
Male, n (%)	3 (75%)	1,229 (68.2%)
Weight (kg), mean (SD)	78.75 (10.3)	78.73 (20.1)
Indication		
Non-Trauma / Medical	1 (25%)	1,013 (56.3%)
Trauma	3 (75%)	788 (43.7%)
After hours (18:00–08:00)	4 (100%)	1,022 (56.8%)
Predicted difficulty	3 (75%)	625 (34.7%)
Checklist used	2 (50%)	1,488 (82.6%)
GCS		
3–8	1 (25%)	1,059 (58.8%)
9–13	1 (25%)	406 (22.5%)
14–15	2 (50%)	336 (18.7%)
RR (breaths/min), median (IQR)	15 (6–23)	18 (13–22)
SBP (mmHg), median (IQR)	129 (49–165)	135 (114–160)
HR (beats/min), median (IQR)	101 (50–126)	95 (76–114)
SpO2 (%), median (IQR)	96 (81–98)	100 (97–100)

Table 2 compares data on preparation for and performance of airway management.

Preoxygenation was attempted in all 4 surgical airway cases, however only 25% received some form of apnoeic oxygenation as compared with 71.7% of the overall cohort (*p* = 0.23). Muscle relaxant medication was administered to facilitate airway management for all surgical airway cases.

**Table 2:** Airway management details

Variable		Surgical Airway	All intubations
		*n* = 4	*n* = 1801
Preoxygenation			
	BVM	2 (50%)	679 (37.7%)
	BVM + PEEP	1 (25%)	877 (48.7%)
	NRBM	1 (25%)	107 (5.9%)
	NIV		96 (5.3%)
	SGA		42 (2.3%)
Apnoeic Oxygenation			
	Not done	3 (75%)	510 (28.3%)
	NP		612 (34.0%)
	BVM	1 (25%)	598 (33.2%)
	NIV		53 (2.9%)
	SGA		28 (1.6%)
Sedative			
	None	1 (25%)	64 (3.6%)
	Ketamine	1 (25%)	1,028 (57.1%)
	Propofol	1 (25%)	432 (24.0%)
	Thiopentone	1 (25%)	188 (10.4%)
	Fentanyl		9 (1%)
	Other		80 (4.4%)
Relaxant			
	Rocuronium	2 (50%)	1,282 (71.2%)
	Suxamethonium	2 (50%)	478 (26.5%)
	Other		41 (2.3%)
Laryngoscope Type			
	Macintosh		194 (10.8%)
	CMAC	3 (75%)	1,548 (86.0%)
	HAVL		48 (2.7%)
	VAFI		3 (0.2%)
	Other	1 (25%)	8 (0.4%)
Adjunct			
	Bougie	4 (100%)	1,437 (79.8%)
	Stylet		91 (5.1%
	Neither		273 (15.2%)
First-attempt laryngoscopist			
	ED Registrar	1 (25%)	1,056 (58.6%)
	ED Consultant		362 (20.1%)
	Anaesthetic Registrar	3 (75%)	212 (11.8%)
	Anaesthetic Consultant		154 (8.6%)
	ICU Registrar		12 (0.7%)
	ICU Consultant		5 (0.3%)

Table 3 summarises the 4 surgical airway cases. Cricothyroidotomy was the primary airway management technique in the second case where trismus prevented laryngoscopy, this could also be considered a failed attempt at intubation, however the literature definition of an attempt includes the insertion of the laryngoscope blade into the mouth which was not possible in this case.

The remainder occurred following at least two failed attempts at intubation.

Time to successful ventilation ranged from 2 min for the primary surgical airway, where the patient was able to have bag-valve-mask ventilation despite their trismus, to 26 min for the first case, which required two attempts at the surgical airway. During these attempts the patient was able to be ventilated to some degree, with a two-handed BVM technique, although this ventilation was documented as suboptimal by the clinicians involved. All cases were able to be ventilated to some extent whilst awaiting completion of the surgical airway. One patient, who was already in cardiac arrest due to overwhelming sepsis at the time of airway management, died in the ED. The remaining cases went to the operating theatre prior to ICU and survived neurologically intact to hospital discharge.

For the eFONA experience survey there was 100% response rate among the 75 EPs approached. The majority of the respondents were male (69%), and the sample encompassed a range of years of experience with a median years of 8 years (IQR 4–14 yrs, range 0-38yrs). The modal number of surgical airways was zero. No clinician had performed more than 2 surgical airways in Australia (overseas experience not included for generalisability). Only three clinicians had kept a logbook of exact number of intubations performed such that the experience denominator was chosen as ‘number of years as a consultant EP’. Overall, clinicians at this centre had performed 24 surgical airways in a total of 768 years of consultant EP experience, or one surgical airway every 32 years.

**Table 3:** Summary of surgical airway cases

Case	Age (years)	Sex	Weight (KG)	Clinical scenario	Predicted difficulty	Airway Assessment	Checkilst used	TL seniority	Preoygenation	Apnoeic oxygenation	Sedative	Relaxant	1st Attempt	2nd Attempt	3rd Attempt	4th Attempt	Time to ventilation	Outcomes	Survival to discharge
1	47	Male	90	Airway / Inhalational burn	Yes	Nasendoscopy revealed early epiglottic swelling	Yes	ED Registrar	BVM	BVM	Propofol	Rocuronium	Anaesthetic Registrar CMAC GD3 Bougie + Cricoid pressure	Anaesthetic Consultant MacIntosh GD3 Bougie + Cricoid pressure Rescue SGA ineffective	ICU Registrar Scalpel-finger-bougie Rescue BVM partially effective	ICU Consultant Scalpel-finger-bougie	26 minutes	Desaturation SpO2 < 93% RMB intubation	Yes
2	29	Female	65	Streptococcus pyogenes (Group A) sepsis with cardiac arrest on arrival and muscle relaxant resistant trismus	No	No anatomical difficulty anticipated (albeit trismus)	No	ED Registrar	BVM	Nil	Nil	Suxamethonium	ED Registrar Scalpel-finger-bougie				2 minutes	Died in ED	No
3	50	Male	80	Neck and facial trauma with profuse epistaxis leading to respiratory failure	Yes	Major facial bleeding	No	ED Consultant	NRBM	Nil	Thiopentone	Suxamethonium	Anaesthetic Registrar CMAC GD3 Bougie + Cricoid pressure	Anaesthetic Consultant CMAC GD4 Bougie + Cricoid pressure	ED Consultant Scalpel-finger-bougie		4 minutes	Desaturation SpO2 < 93% RMB intubation	Yes
4	54	Male	80	Neck and torso trauma - crushed by machinery	Yes	Concern for larnygeal fracture	Yes	ED Consultant	BVM + PEEP	Nil	Ketamine	Rocuronium	Anaesthetic Registrar CMAC GD4 Stylet + Cricoid pressure	Anaesthetic Consultant CMAC GD4 Bougie + Cricoid pressure	ED Consultant Scalpel-finger-bougie		10 minutes	Desaturation SpO2 < 93% RMB intubation	Yes

## Discussion

The need to perform a surgical airway is rare. In our cohort of clinicians there was one surgical airway performed every 30 years. Moreover, the incidence appears to be decreasing in recent years, which may be related to the widespread use of videolaryngoscopy and airway adjuncts, such as the bougie, improved rescue devices, such as second-generation SGAs, and institutional quality improvement programs.

Although uncommon, eFONA performed in a timely fashion can prevent serious morbidity and mortality due to hypoxic brain injury. Through a formal education program which focuses on continuous quality improvement, and standardisation, to optimise team preparation for airway management, this institution maintains a consistently high first attempt success at laryngoscopy [6, 22]. The further refinement and standardisation of equipment during the COVID pandemic has meant that a ‘Plan D’ pouch including a scalpel and size 6.0 mm endotracheal tube is always included in the ‘kit dump’ which is checked as part of the airway checklist [22].

Since the introduction of the quality improvement program there have been 4 surgical airways performed, all of which were successful. All clinicians who successfully secured a surgical airway had attended one of two cadaveric procedure courses, in which attendees repeatedly perform the steps of a cricothyroidotomy on cadavers under calm conditions to build confidence in the performance of this life-saving intervention. At our institution there are 75 emergency physicians on the consultant roster and, of these, 87% have attended one of these courses. Skill fade is an important consideration when considering high-acuity-low-occurrence (HALO) interventions such as eFONA. A systematic review undertaken by the General Medical Council (UK) concluded that clinical skills do fade over time but that the time period and extent to which this happens is highly variable between skills and between individuals, and may be influenced by factors such as level of expertise and opportunities to practice similar skills [24]. The scalpel-finger-bougie-tube technique is also taught using low fidelity models and this method may be particularly effective for spaced repetition and skill maintenance. Moreover, every airway management episode is an opportunity to palpate the anatomy of the anterior neck and mentally-rehearse the procedural steps needed to perform a cricothyroidotomy.

In terms of the human factors related to the *decision* to initiate a surgical airway, confidence in performing the procedure is an important component and may reduce errors of omission. In our education program we also focus on the terminology used and the resulting mindset of trainees in relation to ‘challenging airways’ (anatomically or physiologically difficult airways). Chief among these is the idea that a surgical airway represents a ‘failure’ of airway management. Clearly a surgical airway is not the preferred option if another, less invasive means of ventilation is possible, but equally, death from hypoxia is an unacceptable outcome and we regularly reiterate that this procedure should be considered a ‘successful’ rescue intervention. Moreover, airway doctors do not work in isolation, and as part of our efforts to rebrand the airway nurse as the ‘airway ally’, this team member is empowered to mandate a discussion of a sequential airway plan for every intubation and to raise concerns contemporaneously should deviations from the plan occur.

### Limitations

The small numbers of surgical airways performed limits the statistical power on which to make recommendations regarding these cases. Another limitation of the study is the self-reported nature of the data entry into the registry, which could have introduced bias as clinicians may have been reluctant to report key elements of airway management episodes, particularly failed attempts at intubations or adverse events. It is unlikely, however, that cases of cricothyroidotomy would have been missed as most of the authors are full-time employees of this institution with an interest in emergency airway management. Regarding the survey data, clinicians were all working in the same ED, and as such, may not be reflective of experience in other geographical locations or critical care specialties.

## Conclusion

In the 8-years since the introduction of an institutional airway registry and quality improvement program approximately 2 in every 1000 intubations resulted in a surgical airway. From the individual perspective, clinicians can expect to perform a surgical airway once every 32 years. Although an uncommon intervention, EPs who may be required to undertake or supervise advanced airway management should ensure they have the skills necessary to perform this life-saving intervention.

## Data Availability

The datasets used and/or analysed during the current study are available from the corresponding author on reasonable request.

## References

[1] Rosenstock CV, Nørskov AK, Wetterslev J, Lundstrøm LH. Emergency surgical airway management in Denmark: a cohort study of 452 461 patients registered in the Danish anaesthesia database. Br J Anaesth. 2016;117(Suppl 1):i75–82. 27468737 10.1093/bja/aew190

[2] Bair AE, Panacek EA, Wisner DH, Bales R, Sakles JC. Cricothyrotomy: a 5-year experience at one institution. J Emerg Med. 2003;24(2):151–6. 12609644 10.1016/s0736-4679(02)00715-1

[3] Alkhouri H, Richards C, Miers J, Fogg T, McCarthy S. Case series and review of emergency front-of-neck surgical airways from the Australian and new Zealand emergency department airway registry. Emerg Med Australas. 2021;33(3):499–507. 33179449 10.1111/1742-6723.13678

[4] Offenbacher J, Nikolla DA, Carlson JN, Smith SW, Genes N, Boatright DH, et al. Incidence of rescue surgical airways after attempted orotracheal intubation in the emergency department: A National emergency airway registry (NEAR) study. Am J Emerg Med. 2023;68:22–7. 36905882 10.1016/j.ajem.2023.02.020

[5] Ali AK, Trottier SJ, Christopher Veremakis JOB. Prevalence of difficult/failed airway: Medical-Surgical intensive care unit (ICU).: 486. Crit Care Med. 2006;34(12):A135.

[6] Groombridge C, Maini A, Olaussen A, Kim Y, Fitzgerald M, Mitra B, et al. Impact of a targeted bundle of audit with tailored education and an intubation checklist to improve airway management in the emergency department: an integrated time series analysis. Emerg Med J. 2020;37(9):576–80. 32554746 10.1136/emermed-2019-208935

[7] Fogg T, Alkhouri H, Vassiliadis J. The Royal North shore hospital emergency department airway registry: closing the audit loop. Emerg Med Australas. 2016;28(1):27–33. 26558553 10.1111/1742-6723.12496

[8] von Hellmann R, Fuhr N, Ward A, Maia I, Gerberi D, Pedrollo D, Bellolio F, et al. Effect of Bougie use on First-Attempt success in tracheal intubations: A systematic review and Meta-Analysis. Ann Emerg Med. 2024;83(2):132–44. 37725023 10.1016/j.annemergmed.2023.08.484

[9] Prekker ME, Driver BE, Trent SA, Resnick-Ault D, Seitz KP, Russell DW, et al. Video versus direct laryngoscopy for tracheal intubation of critically ill adults. N Engl J Med. 2023;389(5):418–29. 37326325 10.1056/NEJMoa2301601 PMC11075576

[10] Chang RS, Hamilton RJ, Carter WA. Declining rate of cricothyrotomy in trauma patients with an emergency medicine residency: implications for skills training. Acad Emerg Med. 1998;5(3):247–51. 9523934 10.1111/j.1553-2712.1998.tb02621.x

[11] Heard AM, Lacquiere DA, Gordon HL, Douglas SG, Avis HJ. A case series of the Royal Perth hospital cannula-first approach in the ‘can’t intubate, can’t oxygenate’ scenario. Anaesth Intensive Care. 2024;52(3):159–67. 38546511 10.1177/0310057X231214548

[12] Timmermann A, Chrimes N, Hagberg CA. Need to consider human factors when determining first-line technique for emergency front-of-neck access. Br J Anaesth. 2016;117(1):5–7. 27207773 10.1093/bja/aew107 PMC4913395

[13] Begley JL, Butson B, Kwa P. The emergency surgical airway. Emerg Med Australas. 2017;29(5):570–5. 28929629 10.1111/1742-6723.12850

[14] Chrimes N, Cook TM. Critical airways, critical Language. Br J Anaesth. 2017;118(5):649–54. 28510743 10.1093/bja/aex075

[15] Crewdson K, Lockey DJ. Needle, knife, or device–which choice in an airway crisis? Scand J Trauma Resusc Emerg Med. 2013;21:49. 23806138 10.1186/1757-7241-21-49 PMC3701488

[16] Langvad S, Hyldmo PK, Nakstad AR, Vist GE, Sandberg M. Emergency cricothyrotomy–a systematic review. Scand J Trauma Resusc Emerg Med. 2013;21:43. 23725520 10.1186/1757-7241-21-43 PMC3704966

[17] Price TM, McCoy EP. Emergency front of neck access in airway management. BJA Educ. 2019;19(8):246–53. 33456898 10.1016/j.bjae.2019.04.002 PMC7807984

[18] Groombridge CJ, Maini A, Ayton D, Soh SE, Walsham N, Kim Y, et al. Emergency physicians’ experience of stress during resuscitation and strategies for mitigating the effects of stress on performance. Emerg Med J. 2022;39(11):839–846. 10.1136/emermed-2021-211280 10.1136/emermed-2021-211280 34907004

[19] Groombridge CJ, Kim Y, Maini A, Smit V, Fitzgerald MC. Stress and decision-making in resuscitation: A systematic review. Resuscitation. 2019;144:115–22. 31562904 10.1016/j.resuscitation.2019.09.023

[20] Frerk C, Mitchell VS, McNarry AF, Mendonca C, Bhagrath R, Patel A, et al. Difficult airway society 2015 guidelines for management of unanticipated difficult intubation in adults†. BJA: Br J Anaesth. 2015;115(6):827–48. 26556848 10.1093/bja/aev371 PMC4650961

[21] Chrimes N. The Vortex: a universal ‘high-acuity implementation tool’ for emergency airway management. Br J Anaesth. 2016;117(Suppl 1):i20–7. 27440673 10.1093/bja/aew175

[22] Groombridge CJ, Maini A, Olaussen A, Kim Y, Fitzgerald M, Smit V. Unintended consequences: the impact of airway management modifications introduced in response to COVID-19 on intubations in a tertiary centre emergency department. Emerg Med Australas. 2021;33(4):728–733. 10.1111/1742-6723.13809 10.1111/1742-6723.13809 PMC8209873 34080299

[23] Fennessy P, Drew T, Husarova V, Duggan M, McCaul CL. Emergency cricothyroidotomy: an observational study to estimate optimal incision position and length. Br J Anaesth. 2019;122(2):263–8. 30686312 10.1016/j.bja.2018.10.003

[24] Oates JL. Skills fade: a review of the evidence that clinical and professional skills fade during time out of practice, and of how skills fade May be measured or remediated. Gen Med Council. 2014. www.gmc-uk.org/about/research/26013.asp

